# Validation of the Standardized Outcomes in Nephrology - Life Participation (SONG-LP) Instrument in People Receiving Dialysis

**DOI:** 10.1016/j.ekir.2026.106608

**Published:** 2026-05-20

**Authors:** Cameron Thomas Burnett, Anastasia Hughes, Allison Jaure, Angela Ju, Chandana Guha, Martin Howell, Karine Manera, Melissa Cheetham, Martin Wilkie, Fiona Loud, Ana Figueiredo, Catherine Cheung, Daniel Schwartz, Helen Hurst, Janine Farragher, Jenny Shen, Rachael L. Morton, Rachael C. Walker, Rajnish Mehrotra, Thyago Moraes, Sarbjit V. Jassal, Armando Teixeira-Pinto, Jonathan C. Craig, David W. Johnson, Yeoungjee Cho

**Affiliations:** 1 Department of Kidney and Transplant Services, Princess Alexandra Hospital, Woolloongabba, Queensland, Australia; 2 Kidney Health Service, Logan General Hospital, Meadowbrook, Queensland, Australia; 3 Faculty of Health, Medicine, and Behavioral Science, University of Queensland, Brisbane, Queensland, Australia; 4 Sydney School of Public Health, The University of Sydney, Sydney, New South Wales, Australia; 5 Centre for Kidney Research, The Children’s Hospital at Westmead, Westmead, New South Wales, Australia; 6 Renal Unit, Sunshine Coast University Hospital, Birtinya, Queensland, Australia; 7 Department of Nephrology, Sheffield Teaching Hospitals NHS Foundation Trust, Sheffield, UK; 8 Kidney Care UK, Alton, UK; 9 School of Health Sciences and Life, Nursing School, Pontificia Universidade Católica do Rio Grande do Sul, Porto Alegre, Brazil; 10 Department of Nephrology, Fraser Health, New Westminster, British Columbia, Canada; 11 Division of Nephrology, Department of Medicine, University of British Columbia, Vancouver, British Columbia, Canada; 12 School of Health and Society, University of Salford, UK; 13 Northern Care Alliance NHS Foundation Trust, UK; 14 Department of Occupational Science and Occupational Therapy, University of Toronto, Ontario, Canada; 15 Division of Nephrology and Hypertension, The Lundquist Institute at Harbor-UCLA Medical Center, Torrance, California, USA; 16 NHMRC Clinical Trials Centre, University of Sydney, New South Wales, Australia; 17 School of Nursing, University of Auckland, Auckland, New Zealand; 18 Division of Nephrology, Department of Medicine, Kidney Research Institute, University of Washington, Seattle, Washington, USA; 19 Post-Graduate Program in Health and Biological Sciences, School of Medicine, Pontifícia Universidade Católica do Paraná, Brazil; 20 Division of Nephrology, Department of Medicine, University Health Network, University of Toronto, Ontario, Canada; 21 College of Medicine and Public Health, Flinders Health and Medical Research Institute, Flinders University, Adelaide, Australia; 22 Australasian Kidney Trials Network, University of Queensland, Brisbane, Queensland, Australia; 23 Centre for Kidney Disease Research, University of Queensland, Brisbane, Queensland, Australia; 24 Translational Research Institute, Brisbane, Australia

**Keywords:** core outcome, dialysis, hemodialysis, life participation, peritoneal dialysis, psychometric evaluation

## Abstract

**Introduction:**

Life participation (LP) has been identified as a critically important core outcome for trials in people receiving dialysis. We aimed to validate the Standardized Outcomes in Nephrology-LP (SONG-LP) instrument in people receiving dialysis.

**Methods:**

A psychometric evaluation of the SONG-LP instrument in adults receiving dialysis (including hemodialysis [HD] and peritoneal dialysis [PD]) was completed through a multinational online survey. Internal consistency, test-retest reliability, convergent validity, discriminant validity, and known group comparisons (for dialysis modality and dialysis duration) were assessed.

**Results:**

In total, 250 adults receiving dialysis (69 PD, 47 home HD, and 134 in-center HD) from 13 countries completed surveys at baseline, and 204 participants (82%) completed the survey 1 week later. The SONG-LP instrument demonstrated strong internal consistency (Cronbach’s α = 0.89, 95% confidence interval [CI]: 0.87–0.91, baseline) and test-retest reliability over 1 week (intraclass correlation coefficient [ICC] of 0.76, 95% CI: 0.70–0.81). There was a high correlation (0.76, 95% CI: 0.69–0.81) with the Patient-Reported Outcomes Measurement Information System (PROMIS) Ability to Participate in Social Roles and Activities (APS) Short Form 8a. There was moderate to high correlation with measures assessing concepts related to LP measured by the EuroQol-5 Dimension (EQ-5D) (0.62, 95% CI: 0.53 – 0.69) and PROMIS Cognitive Functional Abilities Subset Short Form 4a (0.49, 95% CI: 0.38 – 0.57).

**Conclusion:**

The SONG-LP instrument is an internally consistent and reliable measure of LP for people receiving dialysis. This study provides preliminary evidence supporting its psychometric validity, offering a foundation for further evaluation of its use in clinical trials.

## Graphical abstract

**Figure:**
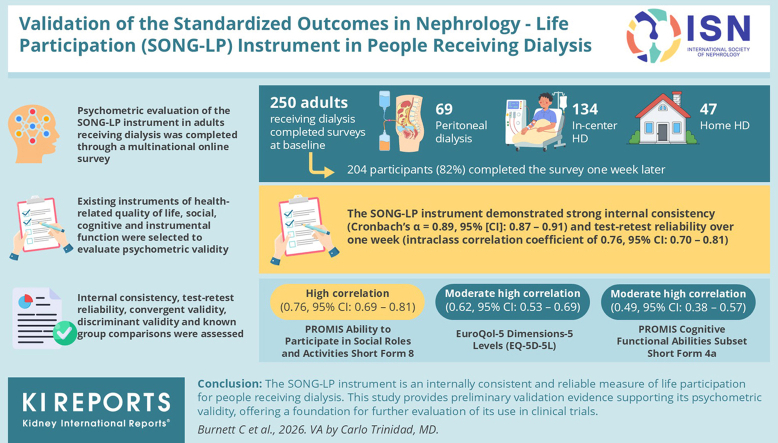

People receiving long-term dialysis have a higher risk of comorbidities and debilitating symptoms, including pain and fatigue, which can significantly impair their quality of life.^1^^,^^2^ For people receiving dialysis, being able to accept and enjoy a life on dialysis is intricately linked to the ability to perform activities that are personally meaningful.^3^^,^^4^ LP, defined as the ability to participate in activities that are meaningful to people, such as work, family, social and leisure activities,^5^ has been identified by the global SONG initiative as a critically important outcome to be reported in all trials for patients with chronic kidney disease before starting kidney replacement therapy (children and adults), and in those receiving PD and kidney transplant receipients.^5^, ^6^, ^7^, ^8^, ^9^ A survey of 100 patients receiving HD highlighted that spending time with friends and family and maintaining ability to work were priority aspects of social wellbeing.^10^ However, LP is rarely and inconsistently assessed in clinical trials involving patients with kidney disease, largely because of the lack of a robust and reliable outcome measures validated for this population.

To address the need for a focused, patient-centered measure, the SONG-LP instrument, a concise 4-item questionnaire (covering ability to participate in activities related to leisure, family, work, and social activities), was developed and validated in kidney transplant recipients, demonstrating simplicity, internal consistency, and reliability.^11^ However, its applicability in patients receiving dialysis has not been demonstrated for implementation. A systematic review of randomized and nonrandomized studies reporting LP in patients on PD identified 42 different measures.^12^ Most were broad health-related quality of life instruments, with LP represented only as a subscale or item.^12^ The SONG-PD LP consensus workshop, which included 56 patients, caregivers, and health professionals from 15 countries, concluded that the SONG-LP instrument captures core domains of LP in a way that remains relevant and specific to each person.^5^ The SONG-LP instrument aligns with the priorities of people receiving dialysis with the following characteristics: (i) brief (<1 minute to complete), thereby reducing survey burden and enhancing dissemination; (ii) codeveloped with patients and caregivers with kidney diseases to ensure meaningful representation of LP compared with other quality of life measures;^13^ and (iii) easy to read, facilitating broader applicability.^14^ This study aimed to assess the psychometric validity of the SONG-LP instrument for use in people receiving long-term dialysis.

## Methods

The psychometric evaluation framework for this study adhered to recommendations from the COnsensus-based Standards for the selection of health Measurement INstruments (COSMIN)-Core Outcome Measures in Effectiveness Trials guidelines.^14^^,^^15^

### Selection and Recruitment of Participants

Participants were eligible to participate if they were > 18 years, currently receiving long-term dialysis (including HD and PD), able to read and write in English, and able to complete online surveys. Participant invitations were widely disseminated through multiple channels, including the SONG-LP expert working groups, social media, local kidney clinics, and patient organizations (i.e., Kidney Health Australia, National Kidney Foundation, Kidney Health New Zealand, and Kidney Care UK). Email invitations were also distributed through the SONG database, which comprises people with chronic kidney disease from 78 countries (38 high-income countries, 37 middle-income, 2 low-income, and 1 unclassified by the World Bank). Human research and ethics committee approval was obtained from the University of Sydney (2021/114) and the University of Queensland (HE002437).

### Data Collection

**Figure 1:**
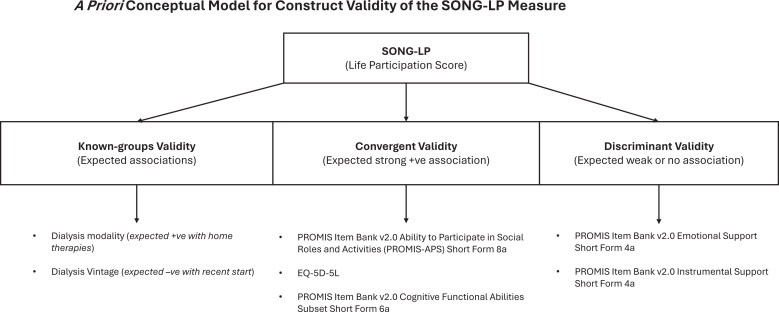
A Priori construct validity framework of the SONG-LP measure. SONG-LP, Standardized Outcomes in Nephrology-Life Participation.

Each participant completed a baseline questionnaire (time point 1) of their demographic and clinical characteristics, and the following 5 measures: SONG-LP, PROMIS Item Bank v2.0 APS Short Form 8a, PROMIS Item Bank v2.0 Cognitive Functional Abilities Subset Short Form 6a, PROMIS Item Bank v2.0 Emotional Support Short Form 4a, and PROMIS Item Bank v2.0 Instrumental Support Short Form 4a. An *a priori* construct validity framework of the SONG-LP measure using these measures is presented in Figure 1. The order of instruments was randomized to minimize the risk of survey ordering bias. One week later (time point 2), participants were asked to complete the SONG-LP instrument and the self-rated kidney function again. The surveys were administered electronically using Research Electronic Data Capture (REDCap),^16^ a secure, web-based software platform designed to support data collection for research studies hosted by institutional servers with encrypted data transmission. The survey was open from 1 February 2025 to 1 July 2025 (Supplementary Table S1).

### Measures

To determine the validity of the SONG-LP instrument, several existing instruments of health-related quality of life, social, cognitive, and instrumental function were selected to evaluate psychometric validity and are detailed below.

#### SONG-LP

**Figure 2:**
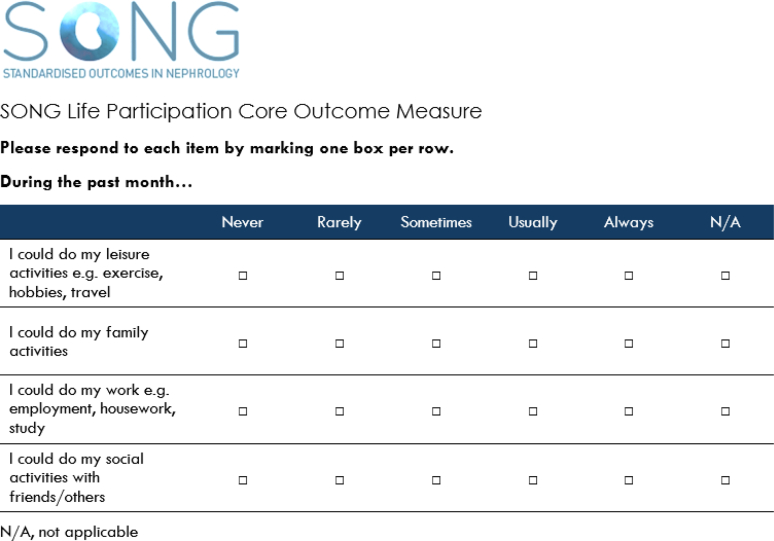
The SONG-LP core outcome measure. SONG-LP, Standardized Outcomes in Nephrology-Life Participation. N/A, not applicable. Adapted with permission from the article by Jaure *et al.*,^11^ Validation of a Core Patient-Reported Outcome Measure for LP in Kidney Transplant Recipients: the SONG-LP Instrument, Figure 1, Copyright Elsevier.

The details of the SONG-LP instrument development have been published previously.^13^^,^^17^ It consists of 4 items rated on a 5-point Likert scale from “never” (0) to “always” (4), with a “not-applicable” (N/A) option. Each item is equally weighted, and the overall score (ranging from 0 to 4) is calculated by averaging available responses, with higher scores indicating better LP (Figure 2). The instrument is categorized as easy to read (Flesch-Kincaid readability grade level 6). The SONG-LP measure has been validated in adult kidney transplant recipients and demonstrated internal consistency, reliability, and convergent validity.^11^

#### PROMIS-APS Short Form 8a

The PROMIS-APS Short Form 8a is an 8-item questionnaire that asks about a person’s perceived difficulty in engaging with their usual social activities, including leisure, family, usual work (i.e., job, housework), and activities with friends.^17^ PROMIS-APS has been validated in people with other severe chronic diseases.^18^^,^^19^ The domains of the PROMIS instrument are similar to the domains assessed by the SONG-LP instrument and were assumed to be positively correlated.

#### EQ-5D-5 Levels

The EQ-5D-5 Levels (EQ-5D-5L) survey is a brief, user-friendly instrument that evaluates health-related quality of life across the following 5 domains: mobility, self-care, usual activities, pain or discomfort, and anxiety or depression.^20^ It includes an item relating to “usual activities” encompassing work, study, housework, family, and leisure, which aligns with the content of the SONG-LP measure of LP and would likely be positively associated with SONG-LP scores. This measure was chosen because of its short length, simplicity, previous validation in both PD^21^ and HD^22^ populations, and utility scores available for different countries to support analyses.^23^

#### PROMIS Item Bank v2.0 Cognitive Functional Abilities Subset Short Form 6a

This 6-item questionnaire assesses impairment of daily functioning, memory, and concentration because of cognitive deficit, which can affect people receiving dialysis.^24^, ^25^ Poorer cognition has demonstrated correlation with low life satisfaction and quality of life. We therefore assumed that this would have a positive correlation with LP.^26^

#### PROMIS Item Bank v2.0 Emotional Support Short Form 4a

People receiving dialysis regularly report feelings of isolation and reduced confidence in relationships.^27^ The PROMIS emotional support instrument directly assesses the perception of feeling valued as an individual and interpersonal security.^28^ As emotional support is inherently different from the ability to participate in life important activities, we presumed this would have no or weak correlation with the SONG-LP measure.

#### PROMIS Item Bank v2.0 Instrumental Support Short Form 4a

This 4-item questionnaire is designed to assess perceived availability of practical help or assistance with day-to-day tasks including chores, transport, mobility, and aid when sick.^29^ We assumed this would have no or weak correlation with the SONG-LP measure.

### Data Analysis

Data analysis was undertaken in R studio (version 2025.09.0, Posit Software PBC, Boston, MA). Categorical variables were summarized as counts and percentages. Demographic and continuous variables were described as medians with interquartile ranges. The scores of different instruments were presented as means and SD. Domains of the SONG-LP measure in people receiving dialysis has been supported through workshops with patients, caregivers, and health professionals.^5^^,^^14^^,^^30^ Construct validity was assessed via convergent validity, acceptability, and hypothesis testing. We assessed reliability by comparing the (a) internal consistency and (b) the test-retest reliability. The following psychometric properties were assessed:

#### Acceptability

Acceptability was evaluated assessing data completeness and response distribution using predefined thresholds of < 5% missing data and < 10% for floor or ceiling effects.^11^

#### Reliability

##### Internal Consistency

Internal consistency was considered acceptable when Cronbach’s alpha was ≥ 0.70, indicating adequate interitem reliability within a unidimensional construct.^15^

##### Test-Retest

To evaluate the test-retest reliability, scores at baseline (time point 1) and at 1 week (time point 2) were compared using ICC, calculated via a 2-way random effects model for single measures. ICC values > 0.75 were interpreted as indicating excellent stability, whereas values between 0.50 and 0.75 reflected moderate stability.^15^^,^^31^

#### Construct Validity

Construct validity refers to the degree to which an instrument accurately measures the theoretical construct it is designed to assess. We evaluated known-groups validity as part of our construct validity assessment. Convergent validity, discriminant validity, and known group comparisons with their hypothesized associations are detailed in Figure 1. Predictive validity and concurrent validity were not evaluated in this study.

##### Convergent Validity

Convergent validity was evaluated using Spearman’s rank correlation coefficients (ρ). Correlation strength was interpreted in accordance with COSMIN-Core Outcome Measures In Effectiveness Trials guidelines as follows: values > 0.7 were considered strong, between 0.3 and 0.7 as moderate, and < 0.3 weak.^15^^,^^32^

##### Discriminant Validity

Discriminant validity was evaluated using Spearman’s rank correlation coefficients (ρ). Consistent with COSMIN-Core Outcome Measures In Effectiveness Trials guidelines, correlations < 0.3 were interpreted as evidence of divergent validity, indicating weak associations with constructs that are theoretically unrelated.^15^^,^^32^

#### Hypothesis Testing

The ability to engage in person-important life activities captured by the SONG-LP measure is likely to be influenced by a person’s dialysis modality (PD, in-center HD, or home HD) and duration of dialysis. It was hypothesized that individuals receiving PD or home HD would report higher SONG-LP scores than those on in-center HD. We hypothesized that individuals with longer exposure to dialysis (i.e., dialysis vintage) would report higher LP scores (Figure 1). Differences in SONG-LP mean scores across dialysis modality or vintage were examined using an independent samples *t* test for 2-group comparisons and 1-way analysis of variance for 3 or more groups. Where assumptions of normality or homogeneity of variance were not met, the nonparametric Kruskal-Wallis test was applied, with *post-hoc* pairwise comparisons to identify specific group differences.

### Sample Size Estimation

A sample size of 250 participants receiving long-term dialysis was required to obtain a precision of 0.06 for the 95% CI for the intraclass correlation coefficient, assuming an ICC of 0.7 for the agreement between the SONG-LP measured 1 week apart.

## Results

In total, 250 participants completed the survey at baseline and 204 (82%) completed the survey after 1 week. Baseline response rate could not be determined because of the open nature of the survey where recruitment was performed using a snowball sampling strategy to improve recruitment of hard-to-reach populations. Participants were from 13 countries, including Australia (*n* = 111, 44%), USA (*n* = 85, 34%), and the UK (*n* = 25, 10%). Eight (3%) participants were from low-middle income countries. Other baseline characteristics are presented in Table 1.

**Table 1:** Demographic and clinical characteristics of participants at baseline (*N* = 250)

Demographic	Median [IQR] or *n* (%)
Age	57 [47, 68]
18–29	15 (6)
30–49	61 (24)
50–69	122 (49)
> 70	52 (21)
Sex	
Male	119 (48)
Female	131 (52)
Country	
Australia	111 (44)
USA	85 (34)
UK	25 (10)
Other^a^	29 (12)
World Bank income group	
High / upper-middle	242 (96.8)
Low / low-middle	8 (3.2)
Marital status	
Married/partner	142 (57)
Separated/divorced	38 (15)
Widowed	14 (6)
Single	55 (22)
Other	1 (< 1)
Employment^b^	
Fulltime	47
Part-time	15
Casual	14
Unemployed	43
Retired	92
Student	4
Other (e.g., disability, carer)	52
Educational attainment	
Primary school (≤ 6 yrs)	0
High school (≤ 12 yrs)	78 (31)
University/higher degree	133 (53)
Other (TAFE/diploma/cert.)	39 (16)
Parental status	
With children	157 (63)
Number of children	2 [2, 3]
Dialysis type	
In-center hemodialysis	134 (53)
Home hemodialysis	47 (19)
Peritoneal dialysis	69 (28)
Dialysis vintage	
< 3 mos	13 (5)
3 to 12 mos	51 (20)
12 to 24 months	49 (20)
> 24 mos	137 (55)
Cause of kidney disease^b^^,^^c^	
Diabetes	42
Glomerular disease	66
Hypertension	64
ADPKD	29
Other^d^	59
Unsure / unknown	40
Comorbidities^c^	
Diabetes	63
Heart disease	57
Cancer	18
Chronic lung disease emphysema	6
Peripheral vascular disease	23
Stroke	9

ADPKD, autosomal dominant polycystic kidney disease; IQR, interquartile range; TAFE, technical and further education.

a Canada (*n* = 6), South Africa (*n* = 6), India (*n* = 5), New Zealand (*n* = 5), Zambia (*n* = 2), Chile (*n* = 1), Spain (*n* = 1), Papua New Guinea (*n* = 1), Puerto Rico (*n* = 1), and Venezuela (*n* = 1).

b Percentages could not be calculated as many participants selected more than 1 option.

c Self-reported.

d Congenital/genetics (*n* = 21), AKI (*n* = 31), and obstructive (*n* = 7).

### Acceptability

The SONG-LP scores were evenly distributed across the scale, without evidence of skewness (Supplementary Figure S1). Floor effects (SONG-LP score = 0) were minimal at both baseline and 1 week (<1%), and ceiling effects (SONG-LP score = 4) were observed in 6% of participants at each time point (predefined acceptability criteria of < 10%). Missing data at 1 week follow-up was 18%, which did not meet the predefined acceptability criteria of < 5% (Supplementary Table S2).

### Reliability

The baseline SONG-LP scores were comparable between those who completed (n = 204) and those who did not complete the survey (n = 46) at 1 week (mean ± SD: 2.30 ± 0.94 vs. 2.48 ± 0.93, *P* = 0.24). Test-retest reliability for the SONG-LP measure demonstrated stability, with an ICC of 0.76 (95% CI: 0.70–0.81). Internal consistency was high with Cronbach’s alpha values of 0.89 (95% CI: 0.87–0.91) at baseline and 0.91 (95% CI: 0.90–0.93) at 1 week (Table 2). By dialysis subgroup, the SONG-LP demonstrated strong internal consistency regardless of dialysis modality, with Cronbach’s α values ranging from 0.87 to 0.90 at baseline and 0.92 to 0.94 at 1 week. Test-retest reliability for each modality was similarly robust using 2-way random sampling intraclass correlation coefficients estimates: in-center HD: 0.85 (95% CI: 0.79 – 0.89); home HD: 0.84 (95% CI: 0.72 – 0.91); PD: 0.89 (95% CI: 0.82 – 0.93; Supplementary Table S3 and S4).

**Table 2:** Mean (SD) scores for SONG-LP and reliability measures (internal consistency and test-retest) and Spearman’s rho correlations between the SONG-LP measure compared with EQ-5D-5L and other PROMIS Item Bank questionnaires in dialysis patients (*N* = 250)

Results	SONG – life participation	PROMIS Item Bank v 2.0				EQ-5D-5L^a^
		APS^a^	CFA^a^	ES^a^	IS^a^	
Mean scores	2.3 (0.9)^a^ 2.3 (1.0)^b^	22.2 (8.0)	21.8 (6.0)	16.6 (4.2)	15.7 (4.6)	0.76 (0.24)
Spearman’s correlation (95% CI)	-	0.76 (0.69–0.81)	0.49 (0.38–0.57)	0.30 (0.17–0.41)	0.22 (0.09–0.34)	0.62 (0.53–0.69)
Cronbach’s α (95% CI)	0.89 (0.87–0.91)^a^ 0.91(0.90 – 0.93)^b^	-	-	-	-	-
ICC (95% CI)^c^	0.76 (0.70–0.81)	-	-	-	-	-

APS, ability to participate in Social Roles and Activities Short Form 8a; CI, confidence interval; CFA, Cognitive Functional Abilities subset Short Form 6a; ES, Emotional Support Short Form 4a; IS, Instrumental Support Short Form 4a; ICC, intraclass coefficient; SONG-L, stSandardized Outcomes in Nephrology- Life Participation.

a Calculated at time point 1.

b Calculated at time point 2.

c Based on 204 participants who completed the survey at 1 week (time point 2).

### Convergent Validity

The SONG-LP instrument demonstrated strong convergent validity with PROMIS-APS with a Spearman’s ρ of 0.76 (95% CI: 0.69 – 0.81). Moderate to strong convergent validity was observed with EQ-5D-5L index scores (ρ = 0.62, 95% CI: 0.53 – 0.69). A moderate correlation was found between SONG-LP scores and the PROMIS - Cognitive Functional Abilities subset Short Form 6a (ρ = 0.49, 95% CI: 0.38 – 0.57, Table 2).

Across dialysis modalities, the SONG-LP instrument showed moderate to strong correlations with PROMIS-APS (ρ = 0.68–0.78) and EQ-5D-5L index scores (ρ = 0.56–0.65), supporting construct validity (Table 3). The PROMIS Cognitive Functional Abilities Short Form 6a was moderately correlated in the in-center HD group (ρ = 0.63), but correlation was attenuated in PD (ρ = 0.26, Supplementary Table S4).

**Table 3:** SONG-LP, EQ-5D-5L, and PROMIS Item Bank scores by dialysis modalities (*N* = 250)

Metric	Dialysis modality		
	In-center HD	Home HD	PD
Participants (*n*)	134	47	69
SONG-LP^a^	2.19 (0.93)^b^ 2.11 (0.90)^c^	2.52 (0.85)^b^ 2.36 (0.98)^c^	2.50 (0.96)^b^ 2.45 (1.05)^c^
APS	21.0 (7.52)	24.1 (7.69)	23.4 (8.6)
CFA	21.4 (6.28)	21.6 (5.55)	22.5 (5.82)
ES	15.9 (4.60)	16.9 (3.81)	17.7 (3.15)
IS	15.1 (4.75)	16.1 (4.59)	16.6 (4.19)
EQ-5D-5L	0.73 (0.24)	0.78 (0.23)	0.82 (0.22)

APS, Ability to Participate in Social Roles and Activities Short Form 8a; CFA, Cognitive Functional Abilities subset Short Form 6a; ES, Emotional Support Short Form 4a; IS, Instrumental Support Short Form 4a; SONG-LP, Standardized Outcomes in Nephrology- Life Participation.

a Statistically significant, *P* = 0.03 (*P* < 0.05).

b Baseline (time point 1).

c One week (time point 2).

### Discriminant Validity

No or weak correlations were observed with the PROMIS Item Bank v2.0 Emotional Support Short Form 4a (ρ = 0.30) and the PROMIS Item Bank v2.0 Instrumental Support Short Form 4a (ρ = 0.22; Table 2). By dialysis subgroup, emotional and instrumental support domains showed weak correlations overall (ρ = 0.06–0.31), with the lowest values in home HD (Supplementary Table S4).

### Hypothesis Testing

#### Dialysis Type

People receiving home therapies (PD and home HD) demonstrated higher LP scores compared with people receiving in-center HD (mean ± SD: 2.51 ± 0.92 vs. 2.19 ± 0.93, *P* = 0.004; Supplementary Figure S2a). When home therapies were stratified by modality, participants receiving home HD and PD reported higher LP scores compared with those receiving in-center HD (2.52 ± 0.85 and 2.50 ± 0.96 vs. 2.19 ± 0.93, *P* = 0.03; Table 3 and Supplementary Figure S2b).

#### Dialysis Vintage

The SONG-LP scores were not statistically significant in the dialysis vintage category (*P* = 0.16; Table 4).

**Table 4:** SONG-LP, EQ-5D-5L and PROMIS Item Bank scores by dialysis vintage (*N* = 250)

Metric	Dialysis vintage			
	< 3 mos	3 – 12 mos	12–24 months	> 24 mos
Participants (*n*)	13	51	49	137
SONG-LP^a^	1.75 (1.14)^b^ 2.03 (0.76)^c^	2.37 (0.94)^b^ 2.24 (1.00)^c^	2.48 (1.02)^b^ 2.43 (1.11)^c^	2.33 (0.87)^b^ 2.22 (0.92)^c^
APS	20.2 (9.45)	23.2 (8.29)	22.3 (8.39)	22.1 (7.56)
CFA	20.8 (5.89)	22.5 (6.67)	22.0 (6.53)	22.1 (7.56)
ES	18.2 (2.38)	16.80 (3.81)	17.3 (3.41)	16.1 (4.59)
IS	15.9 (4.17)	16.2 (4.33)	16.2 (4.30)	15.3 (4.86)
EQ-5D-5L	0.72 (0.22)	0.81 (0.18)	0.77 (0.24)	0.75 (0.26)

APS, Ability To Participate in Social Roles and Activities Short Form 8a; CFA, Cognitive Functional Abilities subset Short Form 6a; ES, Emotional Support Short Form 4a; IS, Instrumental Support Short Form 4a; SONG-LP, Standardized Outcomes in Nephrology-Life Participation.

a *P* = 0.16, *P* > 0.05.

b Baseline (time point 1).

c One week (time point 2).

## Discussion

This study found that the SONG-LP instrument demonstrated acceptability, excellent test-retest reliability, strong convergent validity, and expected discriminant validity as a measure of LP in patients receiving dialysis. The SONG-LP instrument met several reliability and validity criteria outlined by the COSMIN-Core Outcome Measures In Effectiveness Trials guidelines.^15^^,^^32^ Reasonable completion rates (82% at follow-up) and low floor (< 1%) and ceiling (< 5%) effects support its feasibility for implementation. Reliability was demonstrated through high internal consistency and test-retest reliability, exceeding COSMIN thresholds for the Cronbach alpha and ICC.^15^^,^^31^ Construct validity was demonstrated though strong convergent correlation with conceptually related instruments, such as the PROMIS-APS, and a moderate correlation with the EQ-5D-5L and PROMIS-Cognitive Functional Abilities Subset Short Form 6a. There was no association with PROMIS Item Bank v2.0 Emotional Support Short Form 4a, but there was a weak association with the PROMIS Item Bank v2.0 Instrumental Support Short Form 4a, which met the predefined discriminant validity threshold. This likely reflects the fact that these emotional and instrumental support measures do not strongly reflect the domains of LP compared with the PROMIS-APS and EQ-5D-5L. This suggests that perceived LP may be more closely related to independence rather than to perceived support. These psychometric properties were consistent across dialysis modalities, with home-based therapies (home HD and PD) associated with higher SONG-LP scores than in-center HD, supporting our hypothesis that the known-group of home-based therapies would be associated with better LP. Although the score of dialysis vintage was not statistically significant and did not support known-groups validity, the small number of participants within each vintage category limited the ability to examine this adequately. Content validity was established previously through an international SONG consensus workshop to ensure that the SONG-LP instrument included domains of LP that were important to people receiving dialysis.^5^^,^^14^

In comparing patients receiving dialysis with kidney transplant recipients, the SONG-LP instrument demonstrated high internal consistency across both groups (Cronbach’s α ≥ 0.89). However, ceiling effects were substantially lower among dialysis patients (6%) compared with transplant recipients (50%), where many selected “always” across all 4 items.^11^ This difference suggests that the measure may be more sensitive to variations in LP in the dialysis population, where high scores were less common. Average SONG-LP scores were also lower in the dialysis cohort (2.3) compared with the transplant validation cohort (3.1), consistent with previous studies highlighting the greater treatment burden and lifestyle disruption experienced by individuals on dialysis.^11^ Collectively, these findings underscore the utility of SONG-LP in capturing differences in LP across treatment modalities and reflect the substantial challenges faced by people receiving dialysis compared with those living with a functioning kidney transplant.

In this dialysis cohort, strong correlations were observed between SONG-LP and both the PROMIS Item Bank v2.0 APS and the EQ-5D-5L. The correlation with PROMIS-APS was anticipated, as SONG-LP was adapted from this measure and shares similar domains related to LP. The EQ-5D-5L includes a specific item on “usual activities (e.g., work, study, housework, family, or leisure activities),” which likely contributed to its moderate correlation with the SONG-LP. In contrast, low to moderate correlations with other PROMIS measures suggest that these instruments assess different constructs. Interestingly, though most respondents stated that they “always” or “often” had emotional and/or instrumental support, these factors did not correlate with high LP scores. This may reflect 2 possible reasons. First, these measures assess the availability of assistance rather than whether individuals can engage in activities most important to them, which is central to the construct of LP. Second, weaker correlations may indicate that LP is more closely linked to autonomy, symptom burden, and the ability to live independently without reliance on external support, rather than perceived availability of support.

Although the construct of LP in dialysis has not been extensively studied, it is likely to be influenced by a range of clinical and contextual factors over time including, but not limited to, changes in dialysis modality, dialysis dose, access-related complications, hospitalizations and symptom burden. A systematic review and meta-analysis of home dialysis therapies compared with in-center HD demonstrated that health-related quality of life was lower for those receiving in-center HD.^33^ The time since initiation of dialysis was expected to be relevant to LP scores, because it was assumed that LP would improve with time since initiation of dialysis, as a person adapts to treatment-related health behavior changes and the initial disruption to their life. In support of this, health-related quality of life has been previously demonstrated to show gradual improvement following initiation of home-based therapies in a prospective cohort study.^34^ Considering this, we hypothesized *a priori* that LP would differ according to dialysis modality and time since commencing dialysis and used these variables for hypothesis testing and assessment of known-group validity. Differences in SONG-LP scores across dialysis vintages were not statistically significant. However, small subgroup sizes, particularly among patients with less than 3 months on dialysis (*n* = 13), likely resulted in insufficient power to detect meaningful differences.

This study used robust methodology to validate the SONG-LP instrument. Predefined thresholds and hypothesis testing were applied within an international validation cohort from 13 countries, enhancing generalizability of findings. Random allocation of instruments minimized response bias and the high 1 week retention rate (82%) further supports the reliability of results. The inclusion of test-retest reliability is another strength of this study as it is often not measured in psychometric studies. The inclusion of multiple validated comparator instruments strengthened the assessment of construct validity of the SONG-LP instrument. The inclusion of all dialysis modalities meant that differences in LP between modalities could be captured to examine SONG-LP sensitivity to clinical context and patient experience.

Despite these strengths, certain limitations should be acknowledged. The survey was delivered online, self-administered, and in English only, which likely restricted participation to individuals with adequate internet access, digital literacy, and English proficiency. This may have excluded older, frailer, socioeconomically disadvantaged, or culturally and linguistically diverse patients, groups who represent a substantial proportion of the dialysis population and who may have systematically lower LP. Consistent with this, most respondents (97%) were from high-income countries with only 8 respondents (3%) from low- and middle-income countries. Further studies are needed to assess other properties including cultural validity and responsiveness. We acknowledge that because of the open nature of the survey and use of a snowball sampling strategy, the baseline response rate could not be determined. Additionally, there was an 18% attrition in survey response at 1 week, which exceeded our predefined acceptability criteria and increased the likelihood of response bias. The achieved follow-up sample (*n* = 204) was below the prespecified target sample size of 250. Small sample size in the group comparisons analysis (*n* = 13) limited conclusions that can be drawn on this aspect of the instrument’s psychometric validity. In addition, dialysis vintage and comorbidity burden were self-reported, which could have affected the internal validity. Future studies could incorporate validated measures of acceptability, appropriateness, and feasibility, such as the Acceptability of Intervention Measure, Intervention Appropriateness Measure and Feasibility of Intervention Measure to improve researcher confidence in the implementation of this measure.^35^

In conclusion, the SONG-LP instrument demonstrated good reliability and evidence of construct validity in this international cohort of people receiving PD, home HD, and in-center HD. It demonstrates strong internal consistency, construct validity, and sensitivity to differences across dialysis modalities, particularly in capturing social participation. These findings offer a strong basis for further validation, including instrument responsiveness, determination of a minimally important difference, and applicability across culturally and linguistically diverse contexts. Although additional work is required to support the generalizability and suitability of the instrument’s use in dialysis trials, this study highlights the SONG-LP instrument as a promising LP measure for clinical trials aligned with patient-identified priorities.

## Disclosure

TM has received speaker honorarium from AstraZeneca, Bayer, Baxter, Boehringer, CSL Vifor, Libbs, Lilly, Merck, Novo Nordisk, Pfizer, Servier, Takeda, and Vantive. All other authors declared no competing interests with respect to the research, authorship, and/or publication of this article.

## Supplementary Material

Supplementary file (PDF) **Figure S1.** Frequency of SONG LP items scale responses. **Figure S2.** (A) SONG LP scores (In-center versus home therapies). (B) Distribution of SONG LP scores by dialysis modality. **Table S1.** STROBE Checklist for cross-sectional studies. **Table S2.** Data completeness of the SONG life participation measure. **Table S3.** Reliability of SONG-LP by dialysis modality. **Table S4.** Construct validity of SONG-LP by dialysis modality.

## Footnotes

Supplementary file (PDF)
**Figure S1.** Frequency of SONG LP items scale responses. **Figure S2.** (A) SONG LP scores (in-center versus home therapies). (B) Distribution of SONG LP scores by dialysis modality. **Table S1.** STROBE checklist for cross-sectional studies. **Table S2.** Data completeness of the SONG life participation measure. **Table S3.** Reliability of SONG-LP by dialysis modality. **Table S4.** Construct validity of SONG-LP by dialysis modality.
