# Supplementary material for: Validation of the Standardized Outcomes in Nephrology - Life Participation (SONG-LP) Instrument in People Receiving Dialysis
Source: Kidney Int Rep. 2026 May 20;11(7):106608. doi: 10.1016/j.ekir.2026.106608 (PMC13315837; doi:10.1016/j.ekir.2026.106608)
Supplement: Supplementary file (PDF) — Figure S1. Frequency of SONG LP items scale responses. Figure S2. (A) SONG LP scores (In-center versus home therapies). (B) Distribution of SONG LP scores by dialysis modality. Table S1. STROBE Checklist for cross-sectional studies. Table S2. Data completeness of the SONG life participation measure. Table S3. Reliability of SONG-LP by dialysis modality. Table S4. Construct validity of SONG-LP by dialysis modality. [file mmc1.pdf]

Supplementary Files

Supplementary Table S1. STROBE Checklist for cross-sectional studies.
Supplementary Table S2. Data completeness of the SONG life participation measure.
Supplementary Table S3a. Reliability of SONG-LP by dialysis modality.
Supplementary Table S3b. Construct validity of SONG-LP by dialysis modality.
Supplementary Figure S1. Frequency of SONG LP items scale responses.
Supplementary Figure S2a. SONG LP scores (In-centre versus home therapies).
Supplementary Figure S2b. Distribution of SONG LP scores by dialysis modality.

Supplementary Table S1. STROBE Statement for ***cross-sectional studies.***

	Item No	Recommendation	Page No
**Title and abstract**	1	(*a*) Indicate the study's design with a commonly used term in the title or the abstract	Pages 1, 4
		(*b*) Provide in the abstract an informative and balanced summary of what was done and what was found	Page 4
**Introduction**			
Background/rationale	2	Explain the scientific background and rationale for the investigation being reported	Pages 5-6
Objectives	3	State specific objectives, including any prespecified hypotheses	Page 6
**Methods**			
Study design	4	Present key elements of study design early in the paper	Page 6
Setting	5	Describe the setting, locations, and relevant dates, including periods of recruitment, exposure, follow-up, and data collection	Pages 6-7
Participants	6	(*a*) Give the eligibility criteria, and the sources and methods of selection of participants	Page 6
Variables	7	Clearly define all outcomes, exposures, predictors, potential confounders, and effect modifiers. Give diagnostic criteria, if applicable	Pages 6-12
Data sources/ measurement	8*	For each variable of interest, give sources of data and details of methods of assessment (measurement). Describe comparability of assessment methods if there is more than one group	Pages *6-12*
Bias	9	Describe any efforts to address potential sources of bias	Not applicable
Study size	10	Explain how the study size was arrived at	Page 12
Quantitative variables	11	Explain how quantitative variables were handled in the analyses. If applicable, describe which groupings were chosen and why	See 'Data Analysis', Page 10.
Statistical methods	12	(*a*) Describe all statistical methods, including those used to control for confounding	Pages 10-12
		(*b*) Describe any methods used to examine subgroups and interactions	See 'Hypothesis testing' Page 11.
		(*c*) Explain how missing data were addressed	Page 10

		(*d*) If applicable, describe analytical methods taking account of sampling strategy	Not applicable
		(*e*) Describe any sensitivity analyses	Not applicable
**Results**			
Participants	13*	(a) Report numbers of individuals at each stage of study—eg numbers potentially eligible, examined for eligibility, confirmed eligible, included in the study, completing follow-up, and analysed	Page 12, Supplementary Table S2
		(b) Give reasons for non-participation at each stage	Unable to be determined.
		(c) Consider use of a flow diagram	Snowballing recruitment strategy – flow diagram considered unhelpful.
Descriptive data	14*	(a) Give characteristics of study participants (eg demographic, clinical, social) and information on exposures and potential confounders	Table 1 and page 12-13.
		(b) Indicate number of participants with missing data for each variable of interest	Supplementary Table S2.
Outcome data	15*	Report numbers of outcome events or summary measures	Page 12-13.
Main results	16	(*a*) Give unadjusted estimates and, if applicable, confounder-adjusted estimates and their precision (eg, 95% confidence interval). Make clear which confounders were adjusted for and why they were included	Pages 12-14 and Tables 2, 3 and 4. Supp. Tables S3a/b, Supp. Figures 3a/b.
		(*b*) Report category boundaries when continuous variables were categorized	Table 1.
		(*c*) If relevant, consider translating estimates of relative risk into absolute risk for a meaningful time period	Not applicable
Other analyses	17	Report other analyses done—e.g. analyses of subgroups and interactions, and sensitivity analyses	Page 15. Supp. Tables S3a/b, Supp. Figures 2a/b.
**Discussion**			
Key results	18	Summarise key results with reference to study objectives	Pages 12-14.

Limitations	19	Discuss limitations of the study, taking into account sources of potential bias or imprecision. Discuss both direction and magnitude of any potential bias	Pages 18-19.
Interpretation	20	Give a cautious overall interpretation of results considering objectives, limitations, multiplicity of analyses, results from similar studies, and other relevant evidence	Page 19-20.
Generalisability	21	Discuss the generalisability (external validity) of the study results	Page 19.
**Other information**			
Funding	22	Give the source of funding and the role of the funders for the present study and, if applicable, for the original study on which the present article is based	Page 21.

*Give information separately for exposed and unexposed groups.

Note: An Explanation and Elaboration article discusses each checklist item and gives methodological background and published examples of transparent reporting. The STROBE checklist is best used in conjunction with this article (freely available on the Web sites of PLoS Medicine at http://www.plosmedicine.org/, Annals of Internal Medicine at http://www.annals.org/, and Epidemiology at http://www.epidem.com/). Information on the STROBE Initiative is available at www.strobe-statement.org.

Supplementary Table S2. Data completeness of the SONG life participation measure.

Scale	Data completeness (%)	Possible Score Range	Observed Score Range	Mean Score	Standard Deviation	Floor/Ceiling Effect	Skewness
Baseline (TP 1)	250 (100)	0-4	0-4	2.34	0.93	0.01/0.06	-0.20
Week 1 (TP 2)	204 (87)	0-4	0-4	2.26	0.97	0.01/0.06	-0.01

Supplementary Table S3a. Reliability of SONG-LP by dialysis modality.

Metric	In-centre HD<br>α / ICC [95% CI]	Home HD<br>α / ICC [95% CI]	PD<br>α / ICC [95% CI]
**Total participants** (*n*)	134	47	69
**Cronbach's α**	0.90 [0.86–0.92][a]<br>0.90 [0.86–0.92][b]	0.89 [0.83–0.94][a]<br>0.94 [0.90–0.96][b]	0.87 [0.81–0.92][a]<br>0.92 [0.88–0.95][b]
**ICC**	0.85 [0.79–0.89]	0.84 [0.72–0.91]	0.89 [0.82–0.93]

SONG, standardised outcomes in nephrology, ICC = Intraclass correlation coefficient with two-way random effects model, CI = confidence interval.

[a]Time point 1 (baseline)

[b]Time point 2 (1 week)

Supplementary Table S3b. Construct validity of SONG-LP by dialysis modality.

Metric	In-centre HD<br>*ρ [95% CI]*	Home Haemodialysis<br>*ρ [95% CI]*	PD<br>*ρ [95% CI]*
**Total participants** [*n*]	134	47	69
**APS**[c]	0.75 [0.63-0.83]	0.68 [0.46-0.82]	0.78 [0.63–0.87]
**EQ-5D-5L index**	0.59 [0.48-0.70]	0.65 [0.40-0.81]	0.56 [0.37–0.72]
**CFA**[c]	0.63 [0.52-0.71]	0.41 [0.14-0.63]	0.26 [0.01–0.48]
**ES**[c]	0.29 [0.10-0.45]	0.22 [-0.8–0.46]	0.26 [0.00-0.48]
**IS**[c]	0.18 [-0.00-0.36]	0.06 [-0.20-0.34]	0.31 [0.06-0.51]

SONG, standardised outcomes in nephrology, ICC = Intraclass correlation coefficient with two-way random effects model, APS, ability to participate in social roles and activities short form 8a; CFA, cognitive functional abilities subset short form 6a; ES, emotional support short form 4a; IS, instrumental support short form 4a, ρ = spearman's rho. CI = confidence interval.

[a]Time point 1 (baseline)

[b]Time point 2 (1 week)

[c]Patient-Reported Outcomes Measurement Information System (PROMIS) Item Bank v2.0

Supplementary Figure S1. Frequency of SONG LP item scale responses.

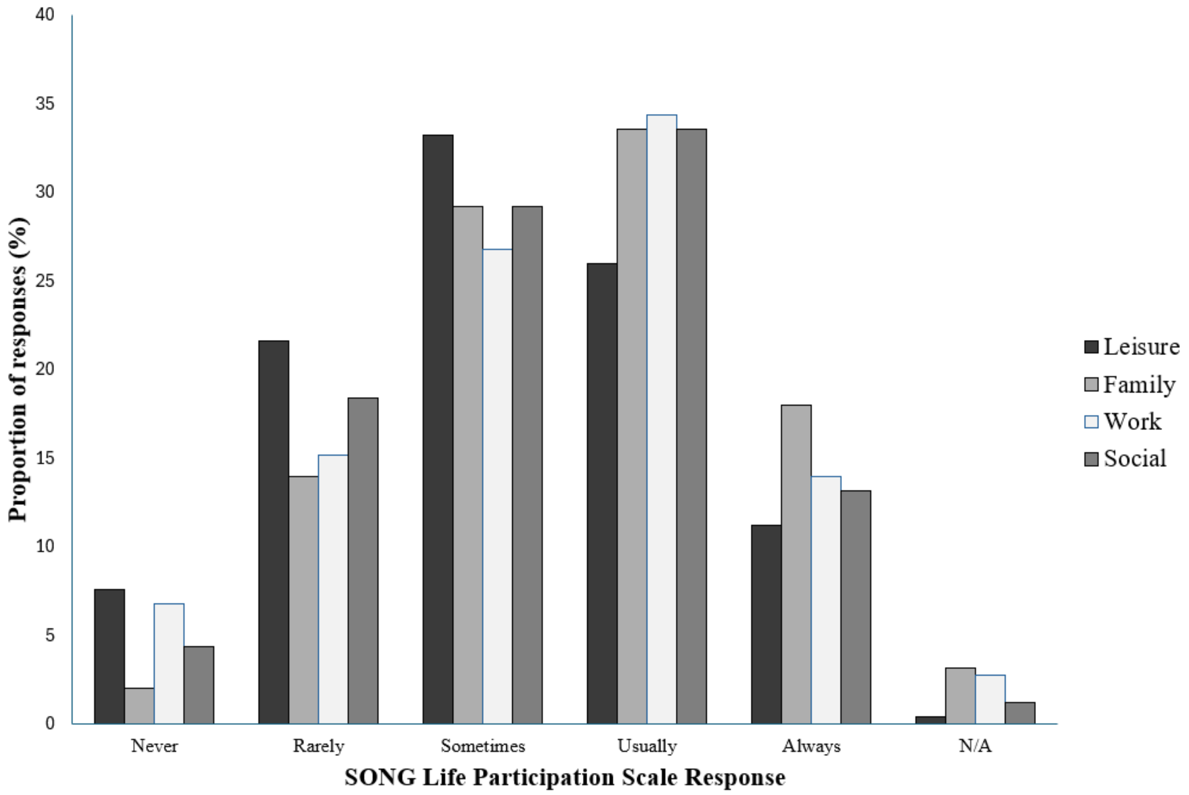

Supplementary Figure S2a. SONG LP scores (In-centre versus home therapies).

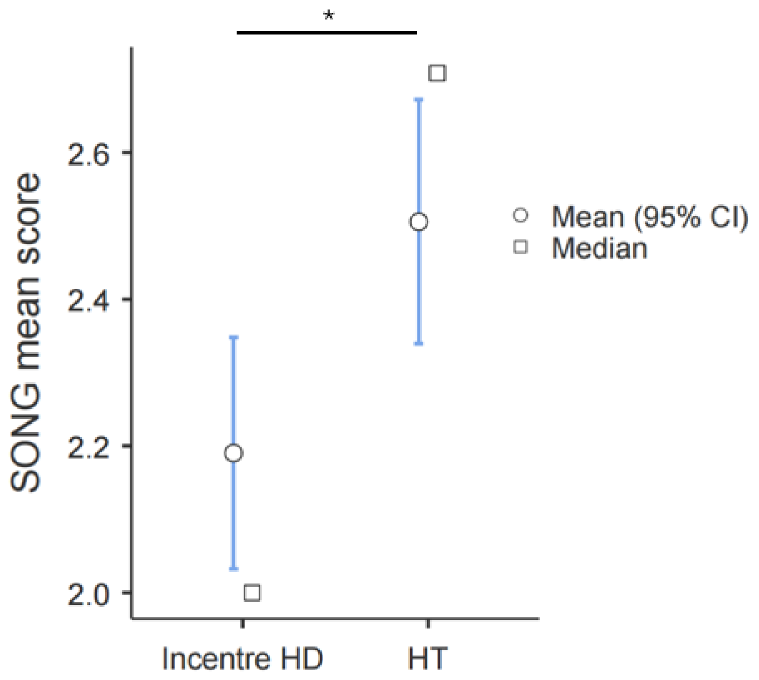

HD = Haemodialysis, HT = Home Therapies. CI = Confidence Interval.

* p-value = 0.004 (Mann-Whitney U test), U = 6219, rank-biserial correlation = 0.20

Supplementary Figure S2b. Distribution of SONG LP scores by dialysis modality.

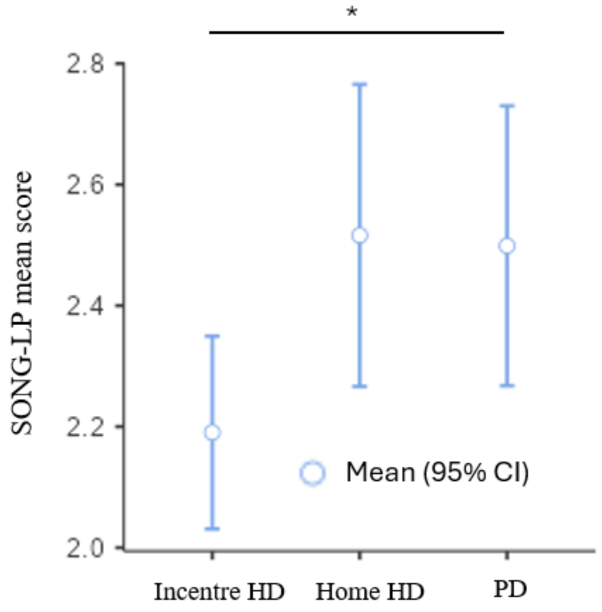

HD = Haemodialysis, PD = Peritoneal Dialysis. CI = Confidence Interval.

*p-value = 0.029 (Kruskal-Wallis 1-way ANOVA).
